# Accuracy of [^18^F]FDG PET/MRI for the Detection of Liver Metastases

**DOI:** 10.1371/journal.pone.0137285

**Published:** 2015-09-03

**Authors:** Karsten Beiderwellen, Llanos Geraldo, Verena Ruhlmann, Philipp Heusch, Benedikt Gomez, Felix Nensa, Lale Umutlu, Thomas C. Lauenstein

**Affiliations:** 1 Department of Diagnostic and Interventional Radiology and Neuroradiology, University Hospital Essen, University of Duisburg-Essen, Essen, Germany; 2 Department of Nuclear Medicine, University Hospital Santa Creu i Sant Pau, Barcelona, Spain; 3 Clinic for Nuclear Medicine, University Hospital Essen, University of Duisburg-Essen, Essen, Germany; 4 Department of Diagnostic and Interventional Radiology, University of Duesseldorf, Duesseldorf, Germany; Kaohsiung Chang Gung Memorial Hospital, TAIWAN

## Abstract

**Background:**

The aim of this study was to compare the diagnostic accuracy of [^18^F]FDG-PET/MRI with PET/CT for the detection of liver metastases.

**Methods:**

32 patients with solid malignancies underwent [^18^F]FDG-PET/CT and subsequent PET/MRI of the liver. Two readers assessed both datasets regarding lesion characterization (benign, indeterminate, malignant), conspicuity and diagnostic confidence. An imaging follow-up (mean interval: 185±92 days) and/-or histopathological specimen served as standards of reference. Sensitivity, specificity, positive predictive value (PPV) and negative predictive value (NPV) were calculated for both modalities. Accuracy was determined by calculating the area under the receiver operating characteristic (ROC) curve. Values of conspicuity and diagnostic confidence were compared using Wilcoxon-signed-rank test.

**Results:**

The standard of reference revealed 113 liver lesions in 26 patients (malignant: n = 45; benign: n = 68). For PET/MRI a higher accuracy (PET/CT: 82.4%; PET/MRI: 96.1%; p<0.001) as well as sensitivity (67.8% vs. 92.2%, p<0.01) and NPV (82.0% vs. 95.1%, p<0.05) were observed. PET/MRI offered higher lesion conspicuity (PET/CT: 2.0±1.1 [median: 2; range 0–3]; PET/MRI: 2.8±0.5 [median: 3; range 0–3]; p<0.001) and diagnostic confidence (PET/CT: 2.0±0.8 [median: 2; range: 1–3]; PET/MRI 2.6±0.6 [median: 3; range: 1–3]; p<0.001). Furthermore, PET/MRI enabled the detection of additional PET-negative metastases (reader 1: 10; reader 2: 12).

**Conclusions:**

PET/MRI offers higher diagnostic accuracy compared to PET/CT for the detection of liver metastases.

## Introduction

Liver metastases substantially influence both prognosis as well as therapeutic options in oncologic patients. An exact localization of liver metastases and the differentiation between unilobar and bilobar metastatic spread is necessary in order to choose the best therapy (liver resection, ablation, chemotherapy)[1–3]. While computed tomography (CT) represents the clinical standard in abdominal tumor staging, magnetic resonance imaging (MRI) of the liver with its excellent soft tissue contrast offers a higher sensitivity especially in liver lesions sized <10 mm[4]. Positron emission tomography (PET) with [^18^F]FDG, usually in combination with CT enables a further characterization of liver lesions based on their glucose metabolism[5]. It allows for an accurate selection of patients with predominantly intrahepatic disease [6], who may benefit from focal therapeutic options. MRI however is known to show a higher sensitivity and accuracy in the detection of small liver lesions compared with CT [7,8] as well as with PET/CT [9], making it is a promising alternative to CT in hybrid imaging. First studies on retrospective fusion of PET and MRI data (referred to as PET/MRI) showed encouraging results [10,11]. However, the quality of co-registration in PET/MRI fusion datasets is not as robust as in co-registered PET/CT data and may be hampered due to different patient positions in PET/CT and MRI [12]. A recent work addressed PET/MRI protocols for liver imaging using a trimodality system (PET/CT and MRI with a shuttle table enabling accurate PET-MRI fusion results)[13].

Only recently, the first integrated PET/MRI scanners allowing for simultaneous data acquisition have been put into operation. Since PET/MRI is still a very young technique the available data regarding its diagnostic value for different applications is preliminary and based on limited patient numbers. First results on performance in different applications in abdominal oncologic applications are promising [14–16]. A comparison on diagnostic accuracy of PET/MRI compared to PET/CT in the detection of abdominal metastases is not available yet. Hence, the aim of this study was to compare the diagnostic accuracy of [^18^F]FDG-PET/MRI with PET/CT for the detection of liver metastases.

## Materials and Methods

**Fig 1:**
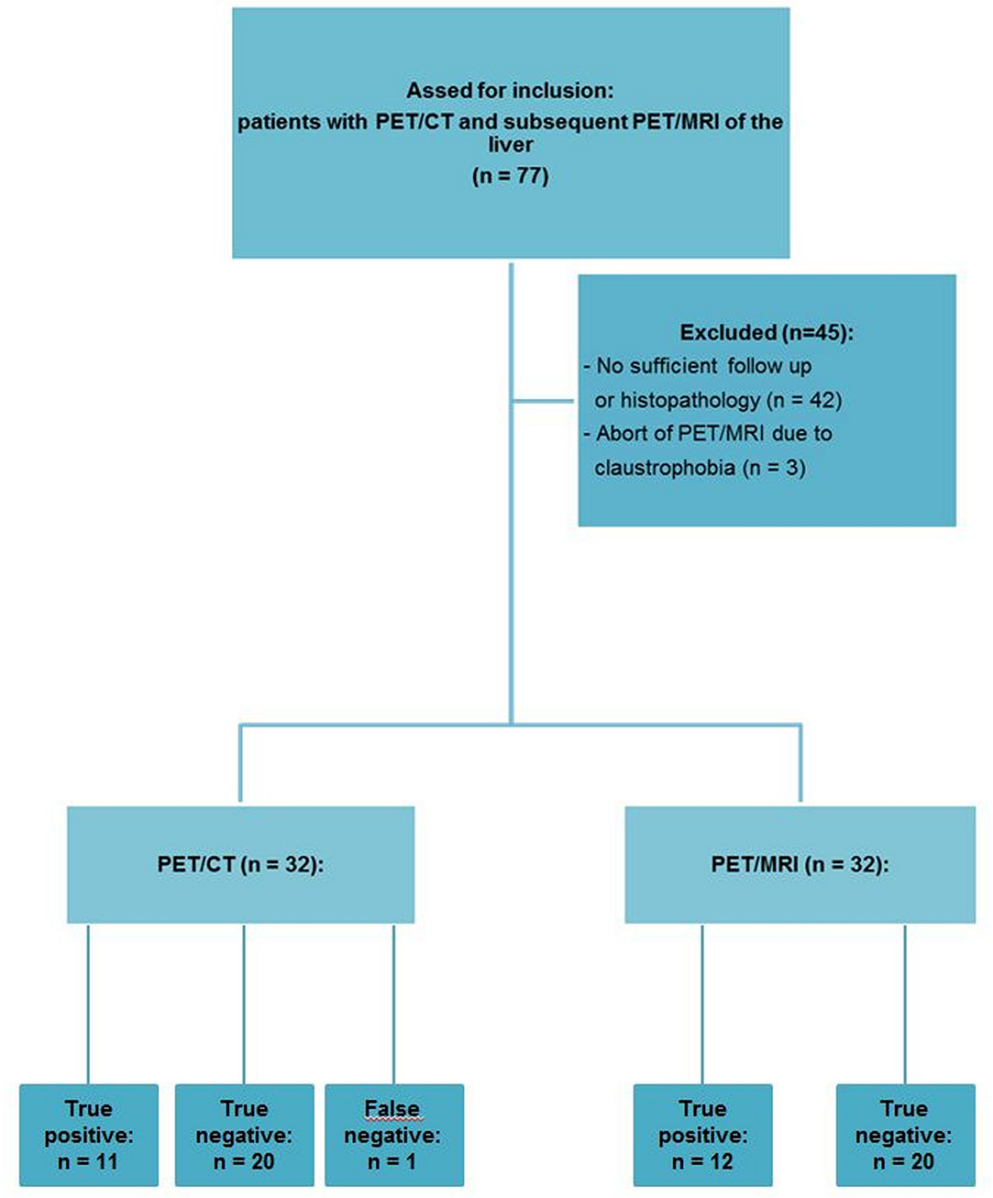
Study flow diagram.

The University Hospital Essen ethics committee approved the examinations as part of fundamental research on integrated PET/MRI. Patient recruitment for PET/CT and PET/MRI was performed between June 2012 and June 2013. 77 consecutive patients underwent with histopathologically confirmed solid malignancies underwent whole body PET/CT with [^18^F]FDG at University Hospital Essen. After written consent all patients were subsequently enrolled for an additional PET/MRI. Inclusion criteria for the study participation comprised age > 18 years, histopathologically confirmed solid malignant disease, a whole body contrast enhanced PET/CT as well as an imaging follow-up of at least 75 days / a histopathological specimen of a target liver lesion. As exclusion criterion renal failure (glomerular filtration rate [GFR] < 30 ml/min) was determined. For the further evaluation three patients were exluded due to an early abort of the PET/MRI due to claustrophobia. 42 patients were exluded due to no sufficient follow-up or the absence of a histopathological sample of a liver lesion (see flow diagram in Fig 1). For the analysis 32 patients (20 women, 12 men, age 57±13 years) were included. Primary tumors included malignant melanoma (n = 7), breast cancer (n = 7), colorectal cancer (n = 4) and others (n = 14).

### Imaging

PET/CT was performed on a Biograph mCT (Siemens Healthare, Erlangen, Germany). CT was acquired using automatic dose modulation at 120 kVp, 210 mAs_eff_, a pitch of 0.8 and a collimation of 128 x 0.6 mm. The scan protocol comprised a pre-contrast liver scan as well as a portal venous phase scan from skull base to mid-thigh with a delay of 70s after administration of 100 ml of iodinated contrast media (Xenetix 300, Guerbet GmbH, Sulzbach, Germany). Patients were positioned supine with elevated arms. PET acquisition was started 60 minutes after i.v.-injection of [^18^F]FDG with a mean activity of 286±45 MBq (range: 222–364 MBq) in 5–6 bed positions (BP) at 2 minutes per BP. 3D PET reconstruction was performed using Ordered Subset Expectation Maximization (OSEM) with 4 iterations and 8 subsets. An image matrix of 256 x 256 and a Gaussian filter with 4.0 mm Full Width at Half Maximum (FWHM) were used. PET attenuation correction was based on portal-venous phase CT.

After PET/CT the patients were transferred to the PET/MRI (Biograph mMR, Siemens Healthcare, Erlangen, Germany). The scan started 148±51 min (range: 101–309 min) after FDG-administration with an acquisition time of 8 min per BP. 3D PET reconstruction was conducted with Ordinary Poisson Ordered Subset Expectation Maximation (OP-OSEM), a matrix of 344 x 344 and a Gaussian filter with 4.0 mm FWHM. A four-compartment-model attenuation map (μ-map) automatically generated based on a two point Dixon sequence was used for attenuation correction. The MR protocol comprised the following sequences:

First, pre- and post-contrast sequences as part of a whole body protocol in free breathing were applied (Sequences A-D and G). Second, dedicated, dynamic contrast enhanced liver series in breath-hold were added (sequences E and F). The detailed MRI-acquisition parameters are displayed in Table 1. Acquisition time for the dedicated PET/MRI liver protocol was 9 min 54 s.

**Table 1:** Acquisition parameters for the applied MR-sequences (A-F).

	A) T1w VIBE Dixon cor.	B) T1w FLASH ax. pre contrast	C) T2w HASTE ax.	D) EPI DWI ax. (b-values: 0, 500, 1000)	E) T2w TSE ax.	F) T1w dyn. VIBE ax.	G) T1w FLASH fs ax. post contrast
**Echo time / Repetition time (ms)**	1.23 + 2.46 (1^st^ + 2^nd^echo) / 3.6	2.15 / 1510	117 / 1500	86 / 11900	97 / 2840	1.51 / 3.75	3.3 / 1700
**Inversion time(ms)**	n/a	1200	n/a	n/a	n/a	n/a	1200
**Flip angle (°)**	10	20	160	90	120	9	20
**Field of view (mm)**	500	450	450	380	400	400	450
**Phase FOV (%)**	65.6	81.3	81.3	75.0	75.0	75.0	81.3
**Parallel imaging acceleration factor**	n/a	2	2	2	2	2	2
**Slice thickness (mm)**	3.1	5.0	5.0	5.0	7.0	3.5	7.5
**Fat saturation (yes/no)**	no	no	no	yes	yes	yes	Yes
**i.v. contrast (yes /no)**	no	no	no	no	no	yes	yes

A) A coronal T1w in and opposed phase Volumetric Interpolated Breath-hold Examination (VIBE) for calculation of the Dixon-based fat-/water-images as well as the μ-map.

B) An axial, pre-contrast T1w Fast Low Angle Shot (FLASH).

C) An axial T2w 2D half Fourier acquisition single shot turbo spin echo (HASTE).

D) An axial diffusion weighted echo planar sequence (EPI DWI) with B values of 0, 500 and 1000 s/mm^2^.

E) An axial T2w fat saturated turbo spin echo (TSE) sequence in breath-hold.

F) A dynamic axial T1w VIBE. Four repetitive scans were performed (pre-contrast, arterial phase [20s delay], portal venous phase [60 sec delay], venous phase [100 sec delay]) after i.v.-injection of 0.1 ml/kg body weight Gadobutrol (Gadovist®, Bayer Healthcare, Berlin, Germany).

G) An axial post-contrast T1w FLASH.

### Data analysis

Two readers with experience in hybrid imaging and MRI of 4 and 6 years, respectively, separately rated the datasets using a viewer software for hybrid imaging (Syngo.via, Siemens Healthcare, Erlangen, Germany). To avoid a possible recall bias the PET/MRI reading session took place two weeks after the PET/CT reading session. For PET/MRI reading the post contrast VIBE was used as anatomic dataset for fusion with the PET dataset. If lesions were only visible in MRI datasets other than the VIBE, they could be fused with the PET dataset by the individual reader.

On a lesion-per-lesion basis up to 10 liver lesions per patient were rated independently by each reader regarding lesion characterization (3 point ordinal scale: 1 = benign, 2 = indeterminate, 3 = malignant), lesion conspicuity (4 point ordinal scale: 0 = not visible, 1 = barely visible, 2 = moderate contrast, 3 = high contrast) and diagnostic confidence (3 point ordinal scale: 1 = uncertain, 2 = rather certain 3 = very certain). For each lesion the affected liver segment was noted. Each lesion was documented by the reader with a screenshot to enable a retrospective analysis. If more than 10 lesions were present, the largest lesions visible in PET/CT and PET/MRI were chosen. In PET-positive lesions (increased tracer accumulation relative to the surrounding liver tissue) the maximum standardized uptake value (SUV_max_) was measured by reader 1 using a spherical volume of interest (VOI).

Since different primary tumors were included that are associated with different contrast behaviors, the raters received information about the primary tumor histology. Criteria for the evaluation of liver lesions in CT included shape, density and contrast enhancement of the lesion. On MRI, lesions were rated as metastases when at least two of the three following criteria were found:

- Hyperintense lesions on T2w images with ill-defined lesion borders
- Diffusion restriction on DWI
- Contrast behavior not in keeping with cysts, hemangioma, focal nodular hyperplasia or adenoma

Lesions detected by PET/MRI that were invisible in the prior PET/CT were reviewed by each reader with regard to visibility in the different MRI sequences.

On a patient basis both readers rated whether liver metastases were present. The evaluation of a possible therapeutic impact was performed using segment related information from the lesion based analysis: based on the described affected liver segments it was determined whether metastases were present in only one liver lobe or if both lobes were affected.

An imaging follow-up of the liver of a minimum of 75 days (185±92 days [range: 77–382]) served as a standard of reference. The imaging modalities of the follow-up examination included PET/CT (n = 13), PET/MRI (n = 1), CT (n = 13), MRI (n = 5). Additionally, histopathological specimens were available in 8 patients (25%).

### Statistical analysis

Data analysis was performed using IBM SPSS for Windows version 20 (IBM Corp., Armonk, NY). A patient-based as well as a lesion based data analysis was performed.

In the lesion based analysis detection rates for each modality were calculated. For this purpose, binary values for lesion characterization were assigned: Lesions rated as benign or indeterminate were assigned 0, lesions rated malignant were assigned 1.

Sensitivity, specificity, positive predictive value (PPV) and negative predictive value (NPV) were calculated using McNemar test. 95% confidence intervals were calculated based on the efficient-score method[17]. A receiver operating characteristic curve was determined for both readers’ ratings separately as well as combined for both PET/CT and PET/MRI. Diagnostic accuracy was assessed by calculating the area under the curve (AUC). Significance of difference in AUCs between the two datasets was determined applying the method by Hanley and McNeal[18,19]. The STARD checklist for accuracy studies is referenced as Supporting Information (S1 File).

Scores for lesion conspicuity and diagnostic confidence of the resulting datasets were analyzed first on a descriptive basis. Median scores were subsequently compared using a non-parametric test (Wilcoxon’s signed rank test). A p-value ≤0.05 was considered to indicate statistical significance. Interobserver agreement of the readers’ ratings for lesions conspicuity, diagnostic confidence and lesion characterization for PET/CT and PET/MRI was determined using Cohen’s ĸ.

In PET-positive lesions SUV_max_ was measured in PET/CT as well as PET/MRI using a spherical volume of interest (VOI). Pearson´s correlation coefficients were calculated to compare SUV_max_ derived from PET/CT and PET/MRI. A p-value ≤0.05 was considered to indicate statistical significance.

## Results

### Patient based analysis

**Fig 2:**
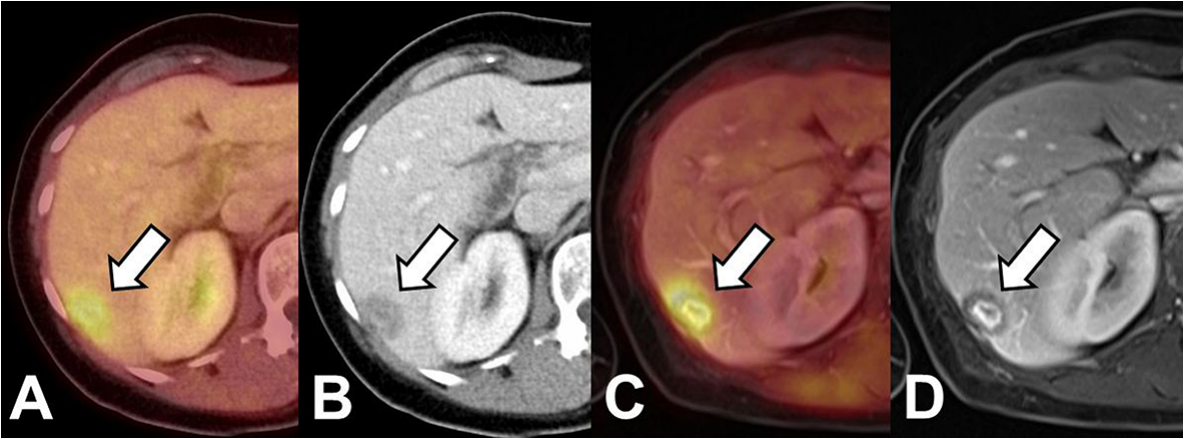
Patient with breast cancer. Both PET/CT (A,B) as well as PET/MRI (C; D; VIBE, portal venous phase) show a lesion with elevated FDG-uptake and ill-defined lesion borders as well as central contrast enhancement as signs of malignancy. Based on these findings the lesion was correctly identified as metastasis in both modalities.

According to the reference standard liver lesions were present in 26 (81%) patients. 12 patients (38%) had liver metastases, benign liver lesions were present in 14 patients (44%). By means of PET/CT 11 of 12 patients with metastases could be identified (92%), whereas PET/MRI rated all 12 disease positive patients as such. Fig 2 displays the case of a patient in which PET/CT as well as PET/MRI correctly identified liver metastases.

**Fig 3:**
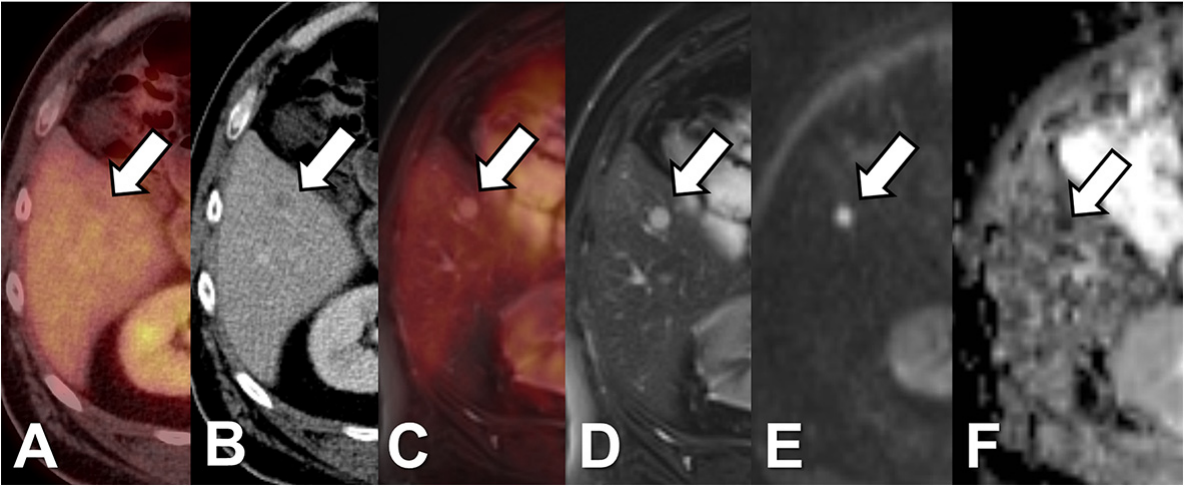
Patient with uveal melanoma. cePET/CT (A+B) shows a hypodense liver lesion of 10 mm in diameter without elevated tracer uptake which is therefore rated as indeterminate. In PET/MRI (C-F) the lesion is hyperintense in T2w TSE (D) and shows signs of restricted diffusion (E: b1000; F: ADC map). Therefore, based on PET/MRI the lesion is rated as metastasis. Based on T2w imaging additional 4 metastases are visualized in PET/MRI in the contralateral lobe.

**Fig 4:**
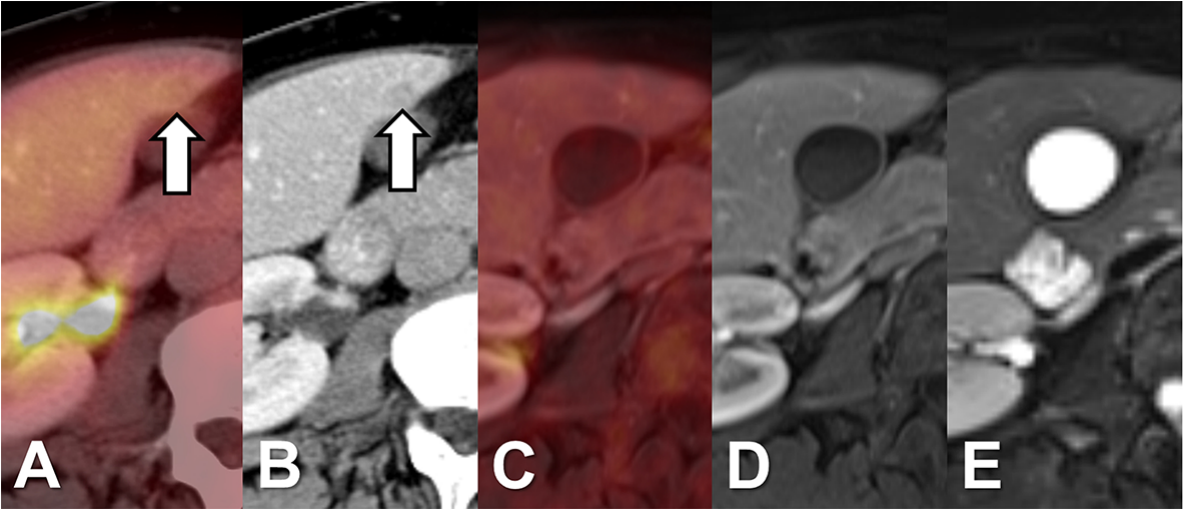
Patient without liver metastases. PET/CT with a false positive result showing a hypodense pseudolesion with a diameter 9 mm in the CT dataset without correlate in PET. In the later acquired PET/MRI (C-E) no correlate in PET nor in the morphological datasets (D: T1w VIBE portal-venous phase; E: T2w TSE fs).

In one patient PET/CT was false negative for a PET-negative hypodense liver metastasis with a diameter of 15 mm, which was rated as a benign lesion. In PET/MRI the lesion equally showed no elevated tracer uptake but exhibited signs of diffusion restriction and was therefore considered as metastasis (Fig 3). Furthermore, 3 additional liver metastases could be identified by PET/MRI in this patient. Follow-up examinations (MRI, last follow-up 441 days) revealed progress in size as well as in the number of lesions. In one patient PET/CT led to a false positive result due a pseudo-lesion in CT of 9 mm without elevated tracer uptake (Fig 4). The corresponding PET/MRI showed no correlate in the MR sequences and no elevated tracer uptake. The follow up examination (PET/CT, interval: 217 days) equally showed no morphological or metabolic correlate.

### Lesion based analysis

According to the standard of reference, a total of 113 liver lesions were found. A mean of 4±3 lesions were present in these patients. Only 2 patients exhibited more than 10 liver lesions. 45 of the 113 lesions (39.8%) were metastases with a mean size of 14±8 mm. 68 of the 113 lesions (60.2%) were benign with an average size of 10±10 mm. A detailed characterization of the benign liver lesions is given in table 2. Of the 45 metastases 26 (58%) showed pathological FDG-uptake. One liver metastasis only showed elevated FDG-uptake in the later acquired PET/MRI. However, this lesion was equally correctly identified as a metastasis in PET/CT due to morphological criteria. FDG-negative liver metastases were significantly smaller than metastases with pathological FDG-uptake (FDG-negative: 11±6 mm; FDG-positive: 17±8 mm; p<0.05).

**Table 2:** Further characterization of benign liver lesions.

	n	%
**Liver cyst**	58	85
**Scar tissue / post-op. changes**	3	4
**Perfusion inhomogeniety**	3	4
**Hemangioma**	2	3
**Focal steatosis**	2	3
**Total**	68	100

For both readers PET/MRI was associated with a significantly higher lesion detection rate (p<0.05). All metastases not detected by PET/CT did not exhibited an elevated FDG-uptake and were less than 10 mm in size (7±2 mm vs. 16±8 mm for metastases detected with PET/CT; p<0.01). The metastases additionally detected by PET/MRI (rater 1: n = 10; rater 2: n = 12) consisted of 8 lesions not visible in PET/CT as well as lesions rated as “indeterminate” in PET/CT (rater 1: n = 2; rater 2: n = 4). All additionally detected metastases in MRI were visualized in the dynamic liver series and DWI (Table 3). Liver metastases not correctly identified by PET/MRI (reader 1: n = 3; reader 2: n = 4) were visible in the MRI datasets but were rated as “indeterminate”. Furthermore, 14 additional benign liver lesions (liver cysts, mean size 4±1 mm) were detected by PET/MRI.

**Table 3:** Liver metastases not visible in PET/CT: detection rate in different MR-sequences.

	Reader 1	Reader 2
T1w FLASH w/o contrast	44% (4/9)	33% (3/9)
T1w FLASH with contrast	44% (4/9)	44% (4/9)
T1w dynamic VIBE	89% (8/9)	89% (8/9)
T2w HASTE	33% (3/9)	33% (3/9)
T2w TSE	67% (6/9)	67% (6/9)
DWI	89% (8/9)	89% (8/9)

Sensitivity, specificity, NPV and PPV for the two readers as well as when combining all ratings by both readers are reported in table 4. PET/MRI showed significantly higher sensitivity values (PET/MRI 92.2%; PET/CT: 67.8%, p<0.01) as well NPV (PET/MRI: 95.1%; PET/CT: 82.0%, p<0.05) whereas no significant difference could be observed for specificity and PPV.

**Table 4:** Sensitivity, Specificity, Accuracy (area under the curve, AUC), PPV, NPV with 95% CI for each reader as well as in combination. Significant differences between PET/CT and PET/MRI are indicated (*: p<0.05; **: p<0.01, ***:p<0.001).

	PET/CT	PET/MRI
**reader 1**		
Sensitivity	71.1% (55.5–83.2%)	93.3% (80.7–98.3%)*
Specificity Accuracy	97.1% (88.8–99.5%)84.1% (75.6–92.6%)	100% (93.3–100%)96.7% (92.4–100%)**
PPV	94.1% (78.9–99.0%)	100% (89.6–100%)
NPV	83.5% (73.1–90.6%)	95.8% (87.3–98.9%)
**reader 2**		
Sensitivity	64.4% (48.7–77.7%)	91.1% (77.9–97.1%)*
Specificity Accuracy	97.1% (88.8–99.5%)80.8% (71.6–89.9%)	100% (93.3–100%)95.6% (90.6–100%)**
PPV	93.5% (77.2–98.9%)	100% (89.3–100%)
NPV	80.5% (70.0–88.1%)	94.4% (85.7–98.2%)
**Overall**		
Sensitivity	67.8% (57.0% - 77.0%)	92.2% (84.1% - 96.5%)**
Specificity Accuracy	97.1% (92.2% - 99.1%)82.4% (76.2–88.7%)	100% (96.6% - 100%)96.1% (92.9–99.4%)***
PPV	93.8% (84.2% - 98.0%)	100% (94.5% - 100%)
NPV	82.0% (75.0% - 87.4%)	95.1% (89.8% - 97.8%)*

**Fig 5:**
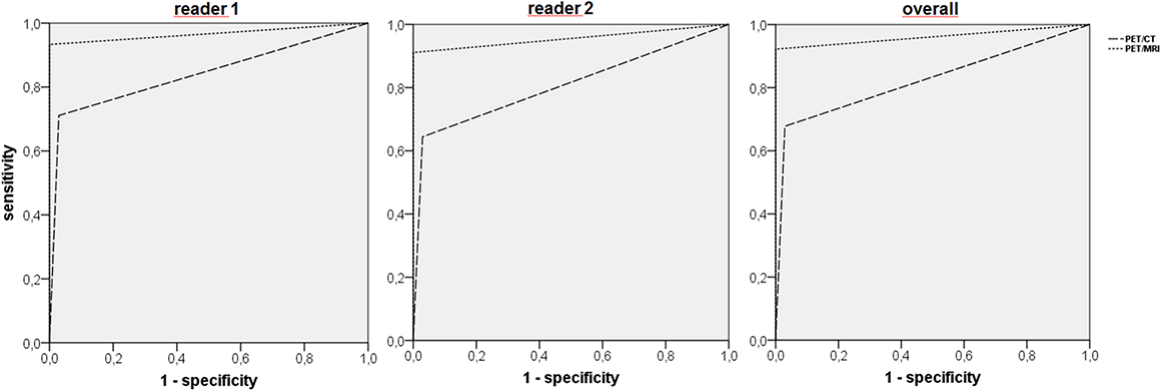
Receiver operating characteristic (ROC) curve for PET/CT and PET/MRI for reader 1 (left), reader 2 (middle) and both readers (right). Significantly higher accuracy (area under the curve, AUC) for PET/MRI (p<0.01 for reader 1 and 2; p<0.001 overall).

Diagnostic accuracy was significantly higher for PET/MRI for both readers (reader 1: PET/CT: 84.1%; PET/MRI: 96.7%; p<0.01; reader 2: PET/CT: 80.8%; PET/MRI: 95.6%; p<0.01) as well as when combining both readers’ ratings (PET/CT: 82.4%; PET/MRI: 96.1%; p<0.001; table 4 and Fig 5).

### Further diagnostic evaluation

Mean SUV_max_ in PET-positive lesions in PET/CT and PET/MRI showed good correlation (PET/CT: 5.9±4.7; PET/MRI: 6.6±5.1; r = 0.88; p<0.001). Lesion conspicuity both for malignant as well as for benign lesions showed a significant difference with higher values for PET/MRI (p<0.001; table 5). Likewise, diagnostic confidence was significantly higher for PET/MRI, both for malignant as for benign liver lesions (p<0.001; table 5). Interobserver agreement was high for lesion conspicuity (PET/CT: k = 0.68; PET/MRI: k = 0.70) and diagnostic confidence (PET/CT: k = 0.63; PET/MRI: k = 0.63) as well as for lesion characterization (PET/CT: k = 0.89; PET/MRI: k = 0.96).

**Table 5:** Lesion conspicuity and diagnostic confidence in PET/CT and PET/MRI. Values are reported as mean±SD [median; range].

	PET/CT	PET/MRI	p-value
**lesion conspicuity**			
** liver metastases**	2.0±1.1[2; 0–3]	2.9±0.8 [3; 1–3]	p<0.001
** benign lesions**	1.9±1.1[2; 0–3]	2.7±0.6 [3, 0–3]	p<0.001
** all lesions**	2.0±1.1[2; 0–3]	2.8±0.5 [3; 0–3]	p<0.001
**diagnostic confidence**			
** liver metastases**	2.3±0.8 [2; 1–3]	2.6±0.7 [3; 1–3]	p<0.001
** benign lesions**	1.9±0.7 [2; 1–3]	2.6±0.6 [3; 1–3]	p<0.001
** all lesions**	2.0±0.8 [2; 1–3]	2.6±0.6 [3; 1–3]	p<0.001

### Therapeutic impact

8 out of 12 patients with liver metastases (67%) had bilobar lesions, whereas 4 patients (33%) showed unilobar disease. All patients with unilobar as well as bilobar metastatic spread were correctly identified by PET/MRI. Based on PET/CT one patient with bilobar PET-negative liver metastases was considered disease-free (Fig 2). Due to the PET/MRI result this patient was upstaged from M0 to M1.

## Discussion

The current study carries two messages we believe to be important: First, PET/MRI as a new diagnostic modality is feasible for an accurate staging with regard to hepatic metastases. Second, it provides a significantly higher diagnostic accuracy in the detection of liver metastases when compared to PET/CT. As a reflection of the higher sensitivity of PET/MRI, more liver metastases can be identified and may even have an impact on therapeutic strategies.

Our results underline the findings of a previous work on PET/MRI of the liver in oncologic patients [15]. As in the present trial PET/MRI offered significantly higher lesion conspicuity as well as diagnostic confidence. In the previous study no additional metastases were detected by PET/MRI. As one major difference to the present study, the MRI was acquired in free breathing without a dynamic contrast enhanced liver series. In spite of a smaller patient cohort more liver metastases were depicted by PET/MRI in the current study. This may not only be related to the additional dynamic liver series (since all metastases were equally identified in DWI), but also owing to the number of considered lesions per patient (5 lesions vs. 10 lesions).

In a recent trial Reiner et al. reported on a study on 55 patients undergoing PET/CT and subsequent MRI of the liver for PET-MRI fusion using a trimodality system[13]. The authors found a high lesion as well as patient based sensitivity (PET/CT: 100%; PET-MRI: 100%) as well as accuracy (PET/CT: 98%; PET-MRI 95%) of PET-MRI. By study design PET/CT was used as standard of reference, a possible superiority of PET-MRI could therefore not be demonstrated. However, the authors added that in PET-MRI using all available MRI sequences additional metastases not detected in PET/CT were found in three patients. Another two patients with bilobar disease were identified using PET/MRI, where PET/CT only identified metastases to one liver lobe. This as well, is in keeping with our results.

In an earlier trial Donati et al. [10] reported on a study of retrospective PET-MRI fusion in 37 patients with suspected liver metastases. All patients underwent PET/CT and subsequent liver MRI with a hepatocyte specific contrast media (Gd-EOB-DTPA). PET-MRI fusion resulted in a significantly higher sensitivity (PET-MRI: 93% PET/CT: 76%, p<0.05) as well as diagnostic accuracy (PET-MRI: 92%- 96%, PET/CT 85%). This is consistent with our findings for accuracy (PET/MRI: 96%, PET/CT: 82%) despite the different acquisition procedures as well as different intravenous contrast media (extracellular vs. hepatocyte specific). However, compared with MRI using hepatocyte specific contrast media, PET-MRI fusion only resulted in a non-significant increase in sensitivity and diagnostic confidence. Moreover, our protocol comprised a DWI sequence, which was not part of the scan procedure by Donati et al. The benefit of liver specific contrast media[20–23], DWI[24,25] as well as the added value of a combination of both[26–29] has already been shown for the detection of liver metastases. It is hypothetical whether a hepatocyte specific contrast phase can even further increase diagnostic accuracy in addition to dynamic contrast enhanced imaging, DWI and metabolic information from [^18^F]FDG-PET.

There are two further aspects of our study that are worthwhile being discussed: First, our results prove inter-rater agreement to be excellent in PET/CT as well as in PET/MRI for lesion conspicuity, characterization and diagnostic confidence. Hence both modalities are not excessively dependent on subjective influences. Second, the use of PET/MRI had considerable impact on the therapy in one patient with a M0 situation according to PET/CT. PET/MRI revealed several small (< 10mm), PET-negative, bilobar liver metastases. This illustrates the benefit of the additional high contrast, high resolution technique of MRI as alternative to CT as morphological modality in hybrid imaging and is in accordance with the findings of Donati et al.[10,13].

Our study is not without some limitations. The patient collective comprised heterogeneous primary tumor entities with metastases exhibiting different appearances in imaging. To overcome this limitation, the readers were not blinded to the primary tumor histology. We did not utilize liver specific contrast media, which is considered to improve sensitivity in the detection of liver metastases[29]. We decided not to use a liver specific contrast agent considering the expenditure of time and a questionable added value in an already high end PET/MRI protocol. It shall be objective of further studies to elucidate whether a liver specific phase can further improve diagnostic accuracy. Due to the study protocol (single injection, double examination) PET/MRI was always performed after a longer injection interval (mean interval: 148min) than PET/CT. Since a longer interval leads to a higher lesion-to-background contrast [30,31], this design might be more favorable for the later acquired PET comprised in PET/MRI. Yet, in our study only one PET-negative metastasis in PET/CT showed elevated FDG-uptake in PET/MRI. However, this lesion could be correctly identified according to morphological criteria in CT. Eventually, histopathological correlation for every depicted lesion was not available due to ethical and practical reasons. However, we overcame this limitation by utilizing a standard of reference encompassing prior exams as well as an imaging follow-up (mean interval: 185±92 days). This form of reference standard has already been successfully used in other trials [32,33].

In conclusion, the results of this study indicate that [^18^F]FDG-PET/MRI is superior to PET/CT for the detection and characterization of liver lesions. Hence, PET/MRI may become a new reference method in oncologic liver imaging as a modality offering a higher diagnostic accuracy at a reduced radiation dose compared to PET/CT.

## Supporting Information

S1 File STARD checklist (STAndards for the Reporting of Diagnostic accuracy studies). (DOC)
