# Supplementary material for: Accuracy of [18F]FDG PET/MRI for the Detection of Liver Metastases
Source: PLoS One. 2015 Sep 3;10(9):e0137285. doi: 10.1371/journal.pone.0137285 (PMC4559465; doi:10.1371/journal.pone.0137285)
Supplement: S1 File — (DOC) [file pone.0137285.s001.doc]

# STARD checklist for reporting of studies of diagnostic accuracy

*(version January 2003)*

| **Section and Topic** | **Item**  **#** |  | **On page #** |
| --- | --- | --- | --- |
| TITLE/ABSTRACT/  KEYWORDS | 1 | Identify the article as a study of diagnostic accuracy (recommend MeSH heading 'sensitivity and specificity').   - *This is stated in the title as well as in the abstract.* - *MesH heading* | 1 and 2 |
| INTRODUCTION | 2 | State the research questions or study aims, such as estimating diagnostic accuracy or comparing accuracy between tests or across participant groups.   - *The research question is stated at the end of the introduction.* | 5 |
| METHODS |  |  |  |
| *Participants* | 3 | The study population: The inclusion and exclusion criteria, setting and locations where data were collected.   - Inclusion criteria comprised age > 18 years, histopathologically confirmed solid malignant disease and a whole body contrast enhanced PET/CT. - As exclusion criterion renal failure (glomerular filtration rate [GFR] < 30 ml/min) was determined. | 6 |
|  | 4 | Participant recruitment: Was recruitment based on presenting symptoms, results from previous tests, or the fact that the participants had received the index tests or the reference standard?   - *Participant recruitment was based on the fact that the participants had received the index* | 6 |
|  | 5 | Participant sampling: Was the study population a consecutive series of participants defined by the selection criteria in item 3 and 4? If not, specify how participants were further selected.   - The study population was a consecutive series of participants defined by the selection criteria. | 6 |
|  | 6 | Data collection: Was data collection planned before the index test and reference standard were performed (prospective study) or after (retrospective study)?   - Data was collection planned before the index test and reference standard (prospective study) | 6 |
| *Test methods* | 7 | The reference standard and its rationale.   - “An imaging follow-up of the liver of a minimum of 75 days (185±92 days [range: 77-382]) served as a standard of reference. The imaging modalities of the follow-up examination included PET/CT (n = 13), PET/MRI (n = 1), CT (n = 13), MRI (n = 5). Additionally, histopathological specimens were available in 8 patients (25%).” - As histopathological sampling is not indicated in the majority of patients with underlying malignant disease (25 % of the patients in this cohort underwent histopathological sampling of liver lesions), the reference standard was complemented by an imaging follow-up. | 10 |
|  | 8 | Technical specifications of material and methods involved including how and when measurements were taken, and/or cite references for index tests and reference standard.   - the technical details for the imaging technique of PET/CT and PET/MRI are given on the pages 6 – 8 | 6 to 8 |
|  | 9 | Definition of and rationale for the units, cut-offs and/or categories of the results of the index tests and the reference standard.   - the definitions for the reading process of PET/CT and PET/MRI are given on the pages 8 to 10. | 8 to 10 |
|  | 10 | The number, training and expertise of the persons executing and reading the index tests and the reference standard.   - “Two readers with experience in hybrid imaging and MRI of 4 and 6 years, respectively, separately rated the datasets using a viewer software for hybrid imaging (…).” | 8 |
|  | 11 | Whether or not the readers of the index tests and reference standard were blind (masked) to the results of the other test and describe any other clinical information available to the readers.   - The readers in the session of the index test (PET/MRI) and reference standard (PET/CT) were blinded the results of the other test. - “Since different primary tumors were included that are associated with different contrast behaviors, the raters received information about the primary tumor histology.” | 8 |
| *Statistical methods* | 12 | Methods for calculating or comparing measures of diagnostic accuracy, and the statistical methods used to quantify uncertainty (e.g. 95% confidence intervals). | 10 |
|  | 13 | Methods for calculating test reproducibility, if done.   - not performed | - |
| RESULTS |  |  |  |
| *Participants* | 14 | When study was performed, including beginning and end dates of recruitment.   - “Patient recruitment for PET/CT and PET/MRI was performed between June 2012 and June 2013.” | 6 |
|  | 15 | Clinical and demographic characteristics of the study population (at least information on age, gender, spectrum of presenting symptoms).   - demographic data as well as type of underlying tumor are stated on page 6 | 6 |
|  | 16 | The number of participants satisfying the criteria for inclusion who did or did not undergo the index tests and/or the reference standard; describe why participants failed to undergo either test (a flow diagram is strongly recommended).   - A flow diagram was included. - We added: “For the further evaluation three patients were exluded due to an early abort of the PET/MRI due to claustrophobia. 42 patients were exluded due to no sufficient follow-up or the absence of a histopathological sample of a liver lesion (see flow diagram in Fig. 1).” | 6 |
| *Test results* | 17 | Time-interval between the index tests and the reference standard, and any treatment administered in between.   - “An imaging follow-up of the liver of a minimum of 75 days (185±92 days [range: 77-382]) served as a standard of reference. The imaging modalities of the follow-up examination included PET/CT (n = 13), PET/MRI (n = 1), CT (n = 13), MRI (n = 5).” - In the consensus reading for the reference standard, the information whether treatment was administered or not in between was available. | 10 |
|  | 18 | Distribution of severity of disease (define criteria) in those with the target condition; other diagnoses in participants without the target condition.   - in the lesion based analysis the number of lesions per patient as well as the size of lesions is given - Furthermore, a discrimination is made between patients with unilobar and bilobar liver metastases - As for benign liver lesions table 2 displays the distribution of benign lesions | 13  + table 2 |
|  | 19 | A cross tabulation of the results of the index tests (including indeterminate and missing results) by the results of the reference standard; for continuous results, the distribution of the test results by the results of the reference standard.   - As in the first revision we were strongly encouraged to cut down on tables, we did not include this additional table and would like to reduce the presented data to table 4 (accuracy, sensitivity, specificity, NPV, PPV). - If necessary however, this additional table can be included | Accuracy:  Table 4, page 29 |
|  | 20 | Any adverse events from performing the index tests or the reference standard.   - There were no adverse events reported neither for the index test nor the reference standard | - |
| *Estimates* | 21 | Estimates of diagnostic accuracy and measures of statistical uncertainty (e.g. 95% confidence intervals).   - These measures are given in table 4 | Table 4 |
|  | 22 | How indeterminate results, missing data and outliers of the index tests were handled.   - Indeterminate results:   - page 10: “In the lesion based analysis detection rates for each modality were calculated. For this purpose, binary values for lesion characterization were assigned: Lesions rated as benign or indeterminate were assigned 0 [not detected], lesions rated malignant were assigned 1 [detected].” - missing data:   - page 6: patients with incomplete examinations were excluded from the study - outliers of the index test:   - there were no outliers in the index test. | 6, 10 |
|  | 23 | Estimates of variability of diagnostic accuracy between subgroups of participants, readers or centers, if done.   - Not performed | - |
|  | 24 | Estimates of test reproducibility, if done.   - Not performed | - |
| DISCUSSION | 25 | Discuss the clinical applicability of the study findings. | 16-19 |
